# Anaphylactic shock with methylprednisolone, Kounis syndrome and hypersensitivity to corticosteroids: a clinical paradox

**DOI:** 10.1186/s13052-018-0579-5

**Published:** 2018-11-28

**Authors:** Nicholas G. Kounis, Ioanna Koniari, George D. Soufras, Emmanouil Chourdakis

**Affiliations:** 1 Department of Cardiology University of Patras Medical School, Rion, 7 Aratou Street, Queen Olgas Square, 26221 Patras, Achaia Greece; 2 Department of electrophysiology, Queen Elizabeth Hospital, Birmingham, England; 3 Department of Cardiology Saint Andrews State General Hospital, Patras, Achaia Greece; 4 Krankenhaus der Barmherzigen Brüder, Trier, Germany

## Abstract

Corticosteroids are widely used for the treatment of allergic reactions but paradoxically themselves may induce acute, delayed, local or systemic allergic reactions and even anaphylaxis with Kounis syndrome. They can suppress the release of arachidonic acid from mast cell membranes, via phospholipase A2 and eicosanoid biosynthesis inhibition. Corticosteroids can promote cell apoptosis and mediate in annexin or lipocortin synthesis, substances that modulate inflammatory cell activation, adhesion molecule expression, transmigratory and phagocytic functions. Antigen-antibody reaction, hapten formation, and medication contaminants are some of the incriminated causes. Patients with atopic diathesis are particularly vulnerable. Complete and thorough previous history of drug reactions or allergies is necessary before administration of any particular medication including corticosteroids.

## To the editor

In the interesting report published in ***Italian Journal of Pediatrics*** [1], an one-year-old male patient suffering from polymalformative syndrome with previous gastrostomy for necrotizing enterocolits and anaphylaxis to cow’s milk proteins, developed repeated episodes of anaphylaxis with urticaria, eyelid edema, tightened laryngospasm and severe dyspnea which occurred within few minutes following intravenous administration of 10 mg of methyl-prednisolone sodium succinate. Skin prick tests and intradermal tests with pure methyl-prednisolone sodium succinate were positive for formulation of 40 mg and negative for formulation of 125 mg. SDS-PAGE (sodium dodecyl sulfate–polyacrylamide gel electrophoresis) and Immunoblotting methods confirmed the presence of allergenic milk proteins in ten different batches of the implicated product (Solu-Medrol 40 mg, Pfizer).

This report raises the following issues on corticosteroid actions and interactions, the immunogenic role of corticosteroid esters and the atopic diathesis-associated immune disorders:

1. Corticosteroids are potent anti-inflammatory and immunomodulating agents which can:
  - Suppress the release of arachidonic acid from mast cell membranes, via inhibition of phospholipase A2, and inhibit eicosanoid biosynthesis [2]
  - Promote cell apoptosis, via autocrine or paracrine pathway involving the up-regulation of the death receptor CD95 and its ligand CD95L [3]
  - Induce annexin or lipocortin synthesis, which modulate inflammatory cell activation, adhesion molecule expression, transmigratory and phagocytic functions [4].
2. Systemic corticosteroids can induce allergic reactions via the following mechanisms:
  - Classical antigen-antibody reactions to corticosteroids and contaminants. Indeed, IgE antibodies to methylprednisolone have been demonstrated in anaphylactic reaction after infusion of methylprednisolone [5].
  - Hapten formation via steroid glyoxal, that is a cortisol degradation product whose aqueous solution could be also responsible for the steroid carbon rings to the body immune system [6].
  - Since the immunogenic role of succinate esters have greater solubility in water and higher affinity to serum proteins, the hapten formation is facilitated and acts as complete antigen [7]
3. Succinate esters of hydrocortisone and methylprednisolone are the drugs that are most frequently responsible for type 1 (immediate) allergic reactions. Indeed, different affinities of hydrocortisone acetate, hydrocortisone sodium phosphate, and hydrocortisone sodium succinate to serum and cutaneous proteins have been demonstrated [8]. However, intravenous administration of 200 mg of hydrocortisone sodium phosphate, for treatment of status asthmaticus [9], has induced itching, macular rash, paresthesiae, tremor of the arms and legs, and nausea and vomiting in 16 patients suffering from chronic asthma.
4. The described patient had experienced anaphylaxis due to cow’s milk proteins administered through gastrostomy when he was 4 months old. Therefore he could be suffered an atopic diathesis. Atopic diathesis involves genetics, immune system dysfunction, environmental exposures, and difficulties with the permeability of the skin. It results from complex abnormalities of the innate and adaptive immune systems. This condition refers to an inherited tendency to produce IgE antibodies in response to small amounts of common environmental factors and atopic patients are easily developed an allergic reaction when they are exposed to such factors. Recent reports have shown that atopic patients suffering from systemic lupus erythematosus have more severe disease at diagnosis and poorer outcomes than SLE patients without atopy [10]. Furthermore, late-onset anaphylactic reactions following intravenous cyclophosphamide pulse in a patient with systemic sclerosis and systemic lupus erythematosus overlap syndrome have been also encountered [11]. Therefore, atopy seems to constitute a disease-substrate that predispose to allergic and anaphylactic reactions. Indeed, a 32-year-old woman suffering from atopic dermatitis [12], without any previous history of food or drug allergy, no suggestive family history, and no other systemic diseases, developed erythematous patches with slight elevation and itching on the face, trunk, and both hands after 0.7 mL of intralesional triamcinolone solution with concentration 2.5 mg/mL on her dorsum of both hands. Her symptoms worsened with therapeutic intravenous re-administration of dexamethasone and she recovered only after treatment with epinephrine
5. In another recent report, a 52-year-old female patient, without previous history of any illness, developed severe retrosternal pain with electrocardiographic ST depression at II, III, aVF, and V3-V6 leads 15 min following 40 mg/ml of intramuscular triamcinolone acetonide (Kenacort-A Retard IM ampule) administration for rashes and itching of her scalp. Troponin was raised (TnI: 0.98 ng/mL), but, on coronary angiography, her coronary arteries were found normal and she recovered with antihistaminic therapy with a final diagnosis of variant type I Kounis syndrome [13]. Additionally, two patients developed anaphylactic shock, with cutaneous and systemic manifestations, following methylprednisolone succinate pulse therapy for neuromyelitis optica and systemic lupus erythematosus [14]. Intradermal skin tests with various types of corticosteroids revealed positive reaction to succinate-containing methyprednisolone, prednisolone, and hydrocortisone in both patients.
6. Furthermore***,*** another 52-year-old woman with a previous history of allergy to anti-haemorrhoid creams and ointments, developed generalized symmetrical pruritic eruption following intra-articular administration of 1 ml of triamcinolone acetonide. Patch tests were positive to tixocortol pivalate, budesonide, hydrocortisone butyrate, nickel sulfate and potassium dichromate. Intradermal skin prick test was positive to triamcinolone and oral challenge tests were positive to deflazacort and methylprednisolone after 14 and 24 h respectively accompanied by pruritic rash. The diagnosis was allergic contact dermatitis progressing to systemic allergic dermatitis [15].

Therefore, physicians should be aware of the possibility of anaphylaxis, allergy or hypersensitivity with corticosteroids. Drugs, including corticosteroids, should be given with caution in patients suffering from comorbid humoral atopic diathesis because such patients seem to have a higher risk of hypersensitivity. Complete and thorough previous history of drug reactions or allergy and clinical examination with prophylactic antiallergic measures including alternative corticosteroids, antihistamines, antiallergic monoclonal antibodies and ingectable epinephrine seem to be of paramount importance.
